# Lecture

**Published:** 1875-04

**Authors:** E. L. Holmes


### Clinics.

### ILLINOIS CHARITABLE EYE AND EAR INFIRMARY.

*Clinical Lectures, by* Dr. E. L. Holmes.

### MALIGNANT DISEASES OF THE EYE.

Gentlemen—I have an opportunity of presenting to you a patient with melanotic cancer of the eye. We can spend an hour no more profitably, I think, than in studying this and the three other cases of malignant disease which have come before us during the last three months.

The patient, an Irish laborer, 50 years of age, gives his history as follows: He was always in excellent health, although thin in flesh, till about six months ago, when he began to lose weight and experience fatigue at ordinary labor. Thirteen years ago he lost the sight of the *left* eye by accident, which caused very great atrophy of the globe and of the corneæ especially. He states that it is but four months since an enlargement of this eye was observed, as also the appearance of numerous small tumors in different parts of the body, which have grown to their present size in this short time.

You now perceive that the patient is exceedingly emaciated, and so weak that he can walk with great difficulty. There are three very large black lobes situated on the upper, outer, and lower portions of the atrophied eye. The mass does not protrude much anteriorly, and yet fills the anterior portion of the orbit so completely that the tip of the finger cannot be pressed between the globe and walls of the orbit. The orbit can scarcely be so completely filled posteriorly as anteriorly, for movements of the sound eye are still accompanied with considerable motion of the diseased eye. I suspect the antero-posterior diameter of the growth is very much less than the perpendicular or transverse diameter.

The patient has experienced a sense of fullness but no pain, although for some weeks he has suffered from a peculiar dizziness. The sclerotic has evidently been absorbed by pressure of the growth from within, which now appears to be covered by a very thin layer of conjunctiva as in certain staphylomata of the choroid and sclerotic. The jet black mass is very hard but not tender on pressure.

On the back, just below the seventh cervical vertebra, is a round tumor, an inch and a half in diameter and movable under the skin. In both gluteal regions, on the left thigh and on the right side of the chest, are similar tumors, some of which are nodulated. On each side of the neck, under the sterno-mastoid muscle, is a large, ill-defined swelling, which is quite firmly attached to the adjacent tissues. These tumors are undoubtedly cancerous.

The iris, ciliary processes and choroid, with all their nerves, have evidently been wholly absorbed, probably long before the commencement of the abnormal growth, for we seldom meet with a case of intra-ocular tumor which has produced so great increase of tension (hardness) without pain.

If the patient was not so positive in regard to the time when he first observed the diseased growths in the eye and other localities, one might almost doubt the correctness of his statement, for I have never met with a case in which the tumor became so large in four months, although I have enucleated the globe for such tumors in fifteen cases. It is impossible to state how long the abnormal process may have existed in the atrophied globe before enlargement began.

There is some obstruction in respiration, possibly induced by cancerous growths in the lungs. The patient is so exhausted that surgical interference is out of the question. (The patient died five days after the clinic, in another institution. No autopsy was permitted.)

This case adds interest to the history of another patient you examined a few weeks ago.

Mr. A. ; forty-seven years of age ; had been blind in the right eye nearly twelve years. During the last two years he suffered frequent attacks of violent pain, with severe symptoms of inflammation. This comprises nearly all the previous history we can obtain. You remember the globe, at our first examination, was *very tense*, the cornea somewhat opaque ; the iris, with pupil closed, appeared to be in contact with the posterior surface of the cornea.

The conjunctiva was greatly congested, although it was not œdematous, neither were the lids swollen.

You recollect I diagnosticated a glaucomatous condition of the globe from choroiditis, possibly caused by the growth of a choroidal sarcoma, and urged the immediate extirpation of the globe. On dividing the globe, after the operation, it was found to be almost entirely filled with a dark colored mass, which the microscope proved to be a sarcoma.

By turning to the specimen which I removed from a young lady two years since, you observe this form of tumor in its early stage, for it occupies but a small portion of the intra-ocular space.

Whenever an adult patient complains of loss of sight in a portion of the field of vision, and you observe with the ophthalmoscope a circumscribed elevation behind the lens, without trembling of the retina, you can almost certainly—according to my experience—diagnosticate the case as some form of tumor, and not a simple detachment of the retina. It is important for you to know that tumors of the choroid, iris, and ciliary bodies, are peculiar to adults, and are almost invariably malignant. The earlier they are removed, by extirpating the globe, the more certainly will life be prolonged.

I now present another unfortunate patient, with an epithelial cancer of the conjunctiva (sclerotic) and cornea. The patient, a farmer, forty years of age, first observed, about eighteen months ago, a peculiar growth near the inner canthus of the left eye. The patient cannot describe its early appearance very satisfactorily, except that at first it was thought to be a pterygium. The growth was removed by his family physician four times during the last four or five months. You now perceive a circular tumor, about half an inch in diameter, and a quarter of an inch in thickness, situated equally on the cornea and sclerotic. The surface is very red and rough, and, except in color, strongly resembles certain fissured warts on the hand. The tumor is remarkably well defined throughout three-quarters of its periphery, which is not the case with the other inner and lower quarter. The conjunctiva adjoining this portion of the tumor, is very red and œdematous, as if the tissue had already invaded it.

Dr. Danforth has kindly examined a minute portion of the tumor and found it to be a typical specimen of epithelial cancer. So well defined is the corneal portion of the tumor that vision is still excellent, although half of the pupil is covered. Most unfortunately the other eye was injured in boyhood to such an extent that vision has ever since been exceedingly imperfect. In enucleating the eye it will be necessary to remove the affected portion of the conjunctiva with the globe.

Before I leave this subject I will exhibit a specimen of melanotic cancer of the conjunctiva (sclerotic) and cornea, which I removed from a lady a year since, and which has a history similar to the one just related, which at first resembled a simple pterygium. You perceive the growth is remarkably well defined, smaller than the one you have just seen, but black and more elevated. The disease had caused considerable pain, which had several times been relieved by removing the tumor. Vision was not impaired.

I think you are safe in assuming that any growth of the sclerotic and cornea, which is firmly attached to the subjacent tissues, and which reappears rapidly after it has been cut away, is malignant. Fortunately doubt can generally be removed by a preliminary microscopic examination of a minute portion of the tumor.

In conclusion, I desire to recall your attention to the two cases of malignant disease in young children, which some of you examined a few weeks since. You observed in an infant twenty months old, a peculiar glistening appearance behind the pupil of the right eye, not much unlike polished German silver. This could be readily seen through the immovable pupil without the aid of the ophthalmoscope. With this instrument vessels could be seen spread over the surface of the tumor. This appearance is so characteristic and so rarely observed except in this disease of the *retina*, called *glioma*, that you can seldom make an incorrect diagnosis in such cases. This disease is most frequently found in young children, and is terribly malignant. Its ultimate development is well illustrated in the case of the boy four years old, which you saw with an enormous fungus protruding from between the distended lids fully an inch and a half, and covered with black crusts.

The removal of glioma by extirpating the whole globe, is generally followed by a speedy development of a similar growth in the orbit, which soon proves fatal. The removal of a sarcoma is a more satisfactory operation, for the cancerous tissue is not especially prone to return in the orbit, but in some of the internal organs. The average duration of life subsequent to such an operation is said to be about four years.

The practical lesson, therefore, for you to learn, is to make an early diagnosis in all these cases, and immediately extirpate the globe.

